# The Role of Distal Medial Cuneiform Angle in Hallux Valgus: A Systematic Review

**DOI:** 10.7759/cureus.102689

**Published:** 2026-01-31

**Authors:** Zain Al Abdeen Al Zuabi, A Aziz, Aakanksha Shetty, Zhikai Li, Rahul Sarda, Alan R Norrish, Chandra Pasapula

**Affiliations:** 1 Medicine and Surgery, The Queen Elizabeth Hospital King's Lynn NHS Foundation Trust, King's Lynn, GBR; 2 Orthopedics, The Queen Elizabeth Hospital King's Lynn NHS Foundation Trust, King's Lynn, GBR; 3 Medicine and Surgery, Addenbrooke's Hospital, Cambridge University Hospitals NHS Foundation Trust, Cambridge, GBR; 4 Academic Unit of Injury, Recovery, and Inflammation Sciences, School of Medicine, University of Nottingham, Nottingham University Hospitals NHS Trust, Nottingham, GBR; 5 Orthopedics and Trauma, The Queen Elizabeth Hospital King's Lynn NHS Foundation Trust, King's Lynn, GBR

**Keywords:** “angulation”, distal medial cuneiform angle, hallux valgus, medial cuneiform, “obliquity”

## Abstract

The obliquity of the first metatarsocuneiform joint has long been proposed as a contributing factor in the development of hallux valgus (HV). Several studies have evaluated the medial slope of the distal articular surface of the medial cuneiform relative to its longitudinal axis, commonly described as the distal medial cuneiform angle (DMCA). An increased DMCA has been hypothesized to predispose individuals to HV by altering first-ray biomechanics.

The objective of this study is to conduct a systematic review of the literature to determine whether DMCA is a significant risk factor for the development of HV.

A systematic literature search was conducted across Ovid, Embase, MEDLINE, and PubMed databases on February 8, 2025, identifying studies published between January 2004 and December 2024. Searches used predefined MeSH terms, including obliquity, angulation, and medial cuneiform, followed by combined-term analysis. English-language full-text articles were screened, and reference lists were reviewed for additional studies. Eligible studies compared DMCA measurements between HV and control cohorts. Where appropriate, pooled effect sizes were calculated using Hedges’ *g*.

The review identified a limited number of heterogeneous observational studies evaluating DMCA in HV populations. Meta-analytic synthesis demonstrated a small but statistically significant association between increased DMCA and HV (Hedges’ *g* = 0.19, *p* = 0.01). However, substantial variability was noted in measurement techniques, radiographic positioning, and definitions of HV severity, limiting comparability across studies.

Current evidence demonstrates a statistically significant but small association between increased DMCA and HV. The modest effect size suggests that DMCA alone is unlikely to be a dominant causal factor and should be considered within a multifactorial biomechanical framework. Further high-quality, standardized studies are required to clarify the clinical relevance of DMCA in the pathogenesis of HV.

## Introduction and background

The obliquity of the first metatarsocuneiform joint has long been considered a potential contributing factor in the development of hallux valgus (HV). Several studies have evaluated the medial slope of the distal articular surface of the medial cuneiform relative to its longitudinal axis, commonly described as the distal medial cuneiform angle (DMCA) [1-5]. An increased DMCA has been theorized to predispose individuals to HV by altering first-ray biomechanics [1].

The medial cuneiform demonstrates considerable anatomical variability and possesses two distal articular surfaces. It has been classified into five distinct morphologic types, such as continuous, bilobed, separated, kidney-shaped, and seven-shaped, and further categorized by facet number as unifacet, bifacet, or trifacet [1]. On weight-bearing anteroposterior radiographs, the distal tarsometatarsal joint (TMTJ) typically exhibits two principal morphological configurations, with Dykyj et al. reporting a more pronounced curvature in women compared with men [6].

It has been proposed that the apex of first-ray deformity in HV is located at the first metatarsocuneiform joint. On this basis, arthrodesis of this joint was first described by Albrecht in 1911 [7] and later popularized by Lapidus in 1934 [8]. A steeper distal articular slope of the medial cuneiform has been hypothesized to contribute to the pathogenesis of HV by promoting medial deviation and instability of the first ray. However, existing evidence directly correlating increased DMCA with greater HV deformity remains inconsistent and inconclusive.

To date, no systematic review has examined whether the DMCA, measured relative to the longitudinal axis of the cuneiform, has been evaluated in relation to its potential causal role in the development of HV. Therefore, the primary hypothesis of this study is that there is no statistically significant difference in DMCA between individuals with HV and those without.

## Review

Aim

This study aimed to conduct a systematic review to determine whether existing literature supports an increase in the DMCA as a significant risk factor for the development of HV.

Methods

A systematic literature search was conducted in Ovid, Embase, MEDLINE, and PubMed databases on February 8, 2025, covering studies published from January 2004 to December 2024.

The final output was then limited to English-language articles with full-text availability. The references from the generated articles were further evaluated for additional studies. All papers were uploaded onto Rayyan software [9], where deduplication and abstract and full-text screening were performed.

Data extraction was performed independently by two reviewers using a predefined data collection template capturing population data and DMCA measurement methods. Extracted data were cross-checked for accuracy and completeness. Any discrepancies were initially resolved through discussion and re-review of the source material; where consensus could not be reached, disagreements were referred to the third senior reviewer (CP), an orthopedic foot and ankle surgeon with over 18 years of experience, for adjudication. This structured approach was used to minimize extraction error and reduce reviewer bias.

The inclusion criteria encompassed all studies relating to the use of the longitudinal axis of the cuneiform to the medial cuneiform slope on the weight-bearing axis (see below).

The search strategy combined both MeSH terms. The following terms were applied: “Obliquity,” “Angulation,” and “Medial cuneiform.”

Initial Search

● “Obliquity” = 11,959 results

● “Angulation” = 62,362 results

● “Medial cuneiform” = 2,579 results

 *Combined Terms*

● Obliquity AND Angulation AND Medial cuneiform = 14

● Obliquity AND Medial cuneiform = 79

● Angulation AND Medial cuneiform = 190

● Obliquity AND Angulation = 777

The combined search resulted in 1,060 records. After applying the inclusion and exclusion criteria, 165 studies remained. After 33 duplicates were removed, 132 records were uploaded into Rayyan for screening.

Inclusion Criteria

● Studies published between January 2004 and December 2024

● Studies conducted on human subjects

● Full-text articles available

● Studies written in the English language

Exclusion Criteria

● Studies on animals

● Articles not written in English or without an available translation

● Letters, editorials, and narrative reviews

● Cadaver-based studies, CT-based studies, non-weight-bearing studies, and finite element modeling studies.

● Studies that did not use the longitudinal axis of the cuneiform as a reference to standardize slope.

The demographic data, including age, sex, and male-female ratio, were also attained.

Hedges' g was used to determine the effect size, as the sample size was small. A forest plot was performed to compare the overall effect size to the line of no effect (set at 0). The IBM Statistical Package for the Social Sciences (SPSS) version 29.0.1.0 (IBM Corp., Armonk, NY) was used to calculate Hedges' g.

Study Selection

**Figure 1:**
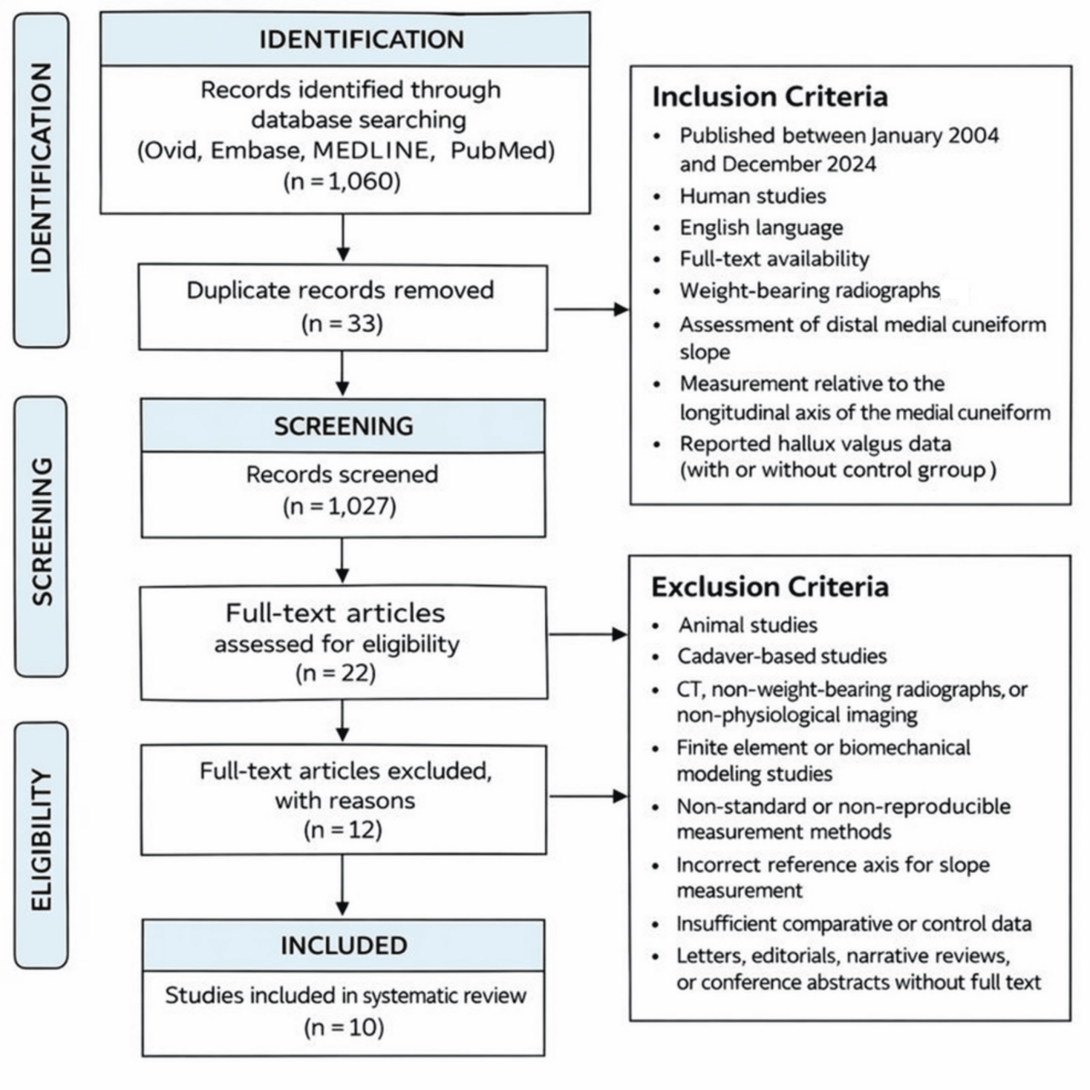
PRISMA 2020 flow diagram illustrating the study selection process for the systematic review PRISMA: Preferred Reporting Items for Systematic reviews and Meta-Analyses.

After Rayyan screened against the eligibility criteria, 110 records were removed. This left 22 full-text articles for detailed review. After full-text screening, 10 studies met all eligibility criteria and were included in the final review (Figure 1).

Results

All eligible studies were independently reviewed, and relevant data were extracted. Studies were included if the distal DMCA was measured using a clearly defined radiographic technique. Three studies (Bu et al., Vyas et al., and Hatch et al. [4,5,10]) reported both HV angles and corresponding control values within matched populations and were therefore included in the primary analysis. Two additional studies reported HV angles but lacked matched control groups and were consequently excluded from the comparative analysis (Table 1).

**Table 1:** Hallux valgus data: DMCA values reported across included studies Values are presented as mean ± standard deviation. Categorical severity subgroups are shown as number of feet with percentage of the study cohort. Studies without a control group are presented without comparative values. DMCA: Distal medial cuneiform angle; HV: Hallux valgus; SD: Standard deviation.

Study	Hallux Valgus Group	Number of Feet, n (%)	Mean Angle (°)	SD (°)	No Hallux Valgus Group	Number of Feet, n (%)	Mean Angle (°)	SD (°)
Erduran et al. [1]	Hallux valgus (overall)	40 (26%)	25.16	5.74	Not reported	—	—	—
	Mild HV	43 (28%)	27.38	6.14	Not reported	—	—	—
	Moderate HV	36 (24%)	30.27	5.62	Not reported	—	—	—
	Severe HV	33 (22%)	34.28	6.81	Not reported	—	—	—
Morio et al. [2]	Mild DCMA	63 (39%)	23.9	6	Not reported	—	—	—
	Moderate HV	36 (22%)	26.4	5.3	Not reported	—	—	—
	Severe HV	64 (39%)	30.2	4.3	Not reported	—	—	—
Bu et al. [4]	Hallux valgus	168 (25%)	21.65	7.93	No hallux valgus	511 (75%)	21.86	7.67
Vyas (Juvenile) et al. [5]	Hallux valgus	46 (56%)	21.66	2.37	No hallux valgus	36 (44%)	22.19	2.23
Hatch et al. [10]	Hallux valgus	25 (50%)	22.27	6	No hallux valgus	25 (50%)	20.68	5.98

One study was excluded because the distal slope was not measured using a standardized or reproducible methodology [11]. Another study was excluded because the reference axis used for slope measurement did not correspond to the lateral border or central longitudinal axis of the medial cuneiform, instead employing a nonstandard variant of the transverse axis [10] (Table 2).

**Table 2:** Study design, population characteristics, and imaging methodology of included studies evaluating distal medial cuneiform angle Values are presented as the number of feet with the percentage of the study cohort, where applicable. This table is descriptive; no statistical comparisons were performed across studies. AP: Anteroposterior; DF: Dorsiflexion; PF: Plantarflexion; HV: Hallux valgus; JHV: Juvenile hallux valgus; PACS: Picture archiving and communication system; WB: Weight-bearing.

Study (Reference)	Year	Study Design	Cohort Type	Sample Size (Feet), n (%)	Sex (M/F), n (%)	Age Range (years)	Imaging Modality	X-ray/Imaging Technique	Study Period
Erduran et al. [1]	2017	Retrospective	Adult patients	152 feet (100%)	Varied by group	16–78	Digital radiographs (PACS)	Weight-bearing AP radiographs	2016–2017
Morio et al. [2]	2021	Retrospective	Adult patients	163 feet (100%)	16 M (17%)/77 F (83%)	≥18	Digital radiographs (PACS)	Weight-bearing AP; beam angled 15° cranial-to-caudal	Apr 2017–Jul 2022
Kaiser et al. (pediatric) [3]	2018	Retrospective comparative	Pediatric patients	143 feet (100%)	Normal: 30% M /70% F; JHV: 25% M/75% F	12–16	Standing radiographs	Weight-bearing AP	2004–2014
Bu et al. [4]	2020	Retrospective	Adult patients	679 feet (100%)	222 M (41%)/316 F (59%)	18–82	Digital radiographs	Weight-bearing AP; beam angled 15°	Jan 2018–Feb 2021
Vyas et al. (pediatric) [5]	2010	Retrospective	Pediatric surgical patients	82 feet (100%)	Not reported	12–16	Radiographs	Pre-operative weight-bearing AP radiographs	1989–2006
Hatch et al. [10]	2016	Prospective comparative	Adult patients	50 feet (100%)	Not reported	≥18	Digital radiographs	Weight-bearing AP; beam angled 15° from coronal plane	Sep 201–Jan 2015
Patel et al. [11]	2019	Cross-sectional	Adult patients	136 feet (100%)	28 M (28%)/71 F (72%)	18–78	Digital radiographs	Standard weight-bearing AP and lateral radiographs	—
Koury et al. [14]	2020	Experimental cadaveric	Cadaveric specimens	10 feet (100%)	5 M (50%)/5 F (50%)	54–88	Fluoroscopic radiographs	Controlled multiplanar positioning (0–20° DF/PF/inversion/eversion)	—
Doty et al. [15]	2014	Experimental cadaveric	Cadaveric specimens	39 feet (100%)	12 M (31%)/22 F (56%)/5 unknown (13%)	45–95	Radiographs + anatomic analysis	Simulated WB AP radiographs with variable beam angulation	—
Li et al. [16]	2024	Case-control	Adult patients	46 feet (100%)	Control: 88% M /12% F; HV: 87% M/13% F	45–70	Computed tomography	Non-weight-bearing CT with 3D reconstruction	—

Three human studies were included in the systematic review. These studies met the inclusion criteria and had quantitative values for both HV feet and controls (feet matched but without HV). Erduran et al. did not have values for HV. Studies such as the one conducted by Morio et al. [2], with no control arm but described DMCA in HV feet only, could not be used as part of the analysis. Kaiser et al. and Patel et al. also used nonstandard methods of measuring slope angle and, therefore, were also excluded from the analysis [3,11].

Overall, there was a higher mean slope for the HV group compared to the non-HV group, with a mean of 26.4 degrees compared to 22.5 degrees in the non-HV group (Table 3).

**Table 3:** Summary of included studies and meta-analysis effect estimates (Hedges’ g)

Study ID	Hedges’ g	Standard Error	95% CI (Lower)	95% CI (Upper)	p-Value	Weight	Weight (%)
Erduran et al. (mild) [1]	0.37	0.22	−0.06	0.8	0.09	20.76	9.75
Erduran et al. (severe) [1]	0.89	0.24	0.42	1.36	<0.001	17.6	8.26
Erduran et al. severe [1]	1.44	0.26	0.93	1.96	<0.001	14.68	6.89
Bu et al. [4]	−0.03	0.09	−0.20	0.15	0.76	126.71	59.49
Vyas et al. (Juvenile) [5]	−0.23	0.22	−0.66	0.21	0.3	20.45	9.6
Hatch et al. [10]	0.26	0.28	−0.29	0.81	0.35	12.79	6.01
Overall	0.19	0.07	0.05	0.32	0.01	—	—

Bu et al. and Vyas et al. [4,5] both demonstrated slightly lower means in HV compared to the non-HV (21.7 degrees). Vyas et al. evaluated juvenile hallux valgus (JHV) patients. Bu et al. had the largest sample size of 168 feet. Hatch demonstrates a slightly higher mean DMCA in the HV group compared to the non-HV group (Table 4).

**Table 4:** Demographic characteristics, cohort composition, and study design of included studies assessing distal medial cuneiform angle Values are presented as numbers with percentage of the study cohort, where applicable. This table is descriptive; no statistical comparisons were performed across studies. AP: Anteroposterior; HV: Hallux valgus; HVA: Hallux valgus angle; JHV: Juvenile hallux valgus; IMA: Intermetatarsal angle; WB: Weight-bearing.

Study	Participants	Feet	Sex (M/F), n (%)	Age (Mean/Range, Years)	Groups and Criteria, n (%)	Inclusion Criteria	Exclusion Criteria	Study Period
Erduran et al. [1]	152 pts	152 feet	Varied by group	Normal 45; Mild 51; Mod 49; Severe 52	Normal 40 (26%); HV 112 (74%)–Mild 43, Mod 36, Severe 33	Normal + mild–severe HV	Congenital anomalies, surgery, fractures, diabetic feet	2016–2017
Morio et al. [2]	93 pts	163 feet	16 M (17%)/77 F (83%)	Mean 68.9	HV only–Mild 63 (39%), Mod 36 (22%), Severe 64 (39%)	Adults ≥ 18	Trauma, prior surgery, inflammatory disease	2024
Kaiser et al. (pediatric) [3]	93 pts	143 feet	Normal: 15 M (30%)/35 F (70%); JHV: 23 M (25%)/70 F (75%)	Normal 13.2; JHV 14.1	Normal 36 pts (50 feet, 35%); JHV 57 pts (93 feet, 65%)	Idiopathic JHV; WB AP XR	Neuromuscular disease, prior surgery, severe deformity	2004–2014
Bu et al. [4]	538 pts	679 feet	222 M (41%)/316 F (59%)	18–82	Normal: 155 pts (168 feet, 25%); HV: 383 pts (511 feet, 75%)	Diagnosis of HV; WB AP radiograph	Congenital deformity, prior surgery, severe arthritis	Jan 2018–Feb 2021
Vyas et al. (pediatric) [5]	54 pts	82 feet	Not reported	Normal 13.2; HV 14.2	Normal 25 (46%) vs HV 29 (54%)	Idiopathic JHV	Gross deformity, underlying pathology	2010
Hatch et al. [10]	50 total	50 feet	Not reported	≥18	Normal: 25 (50%) (IMA < 10°); HV: 25 (50%) – Moderate 15 (60%), Severe 10 (40%)	IMA criteria; no previous 1st-ray surgery	Not reported	Sep 2014–Jan 2015
Patel et al. [11]	99 pts	136 feet	28 M (28%)/71 F (72%)	18–78 (mean 47.4)	Not stratified	Not specified	Not specified	2019
Koury et al. [14]	Cadaveric	10 feet	5 M (50%)/5 F (50%)	54–88 (mean 79.9)	No subgroups reported	Cadaver availability	None reported	Not specified
Doty et al. [15]	Cadaveric	39 feet	12 M (31%)/22 F (56%)/5 unk (13%)	Mean 79 (45–95)	Normal 5 (13%); HV 34 (87%)–Mild 12, Moderate 18, Severe 4	Cadaveric specimens	Prior foot surgery, significant pathology	Not specified
Li et al. [16]	32 pts	46 feet	Control: 15 M (88%)/2 F (12%); HV: 13 M (87%)/2 F (13%)	Control 52.3; HV 55.3	Control (HVA < 15°) vs HV (HVA < 40°)	HV vs normal groups	Not specified	2024

**Figure 2:**
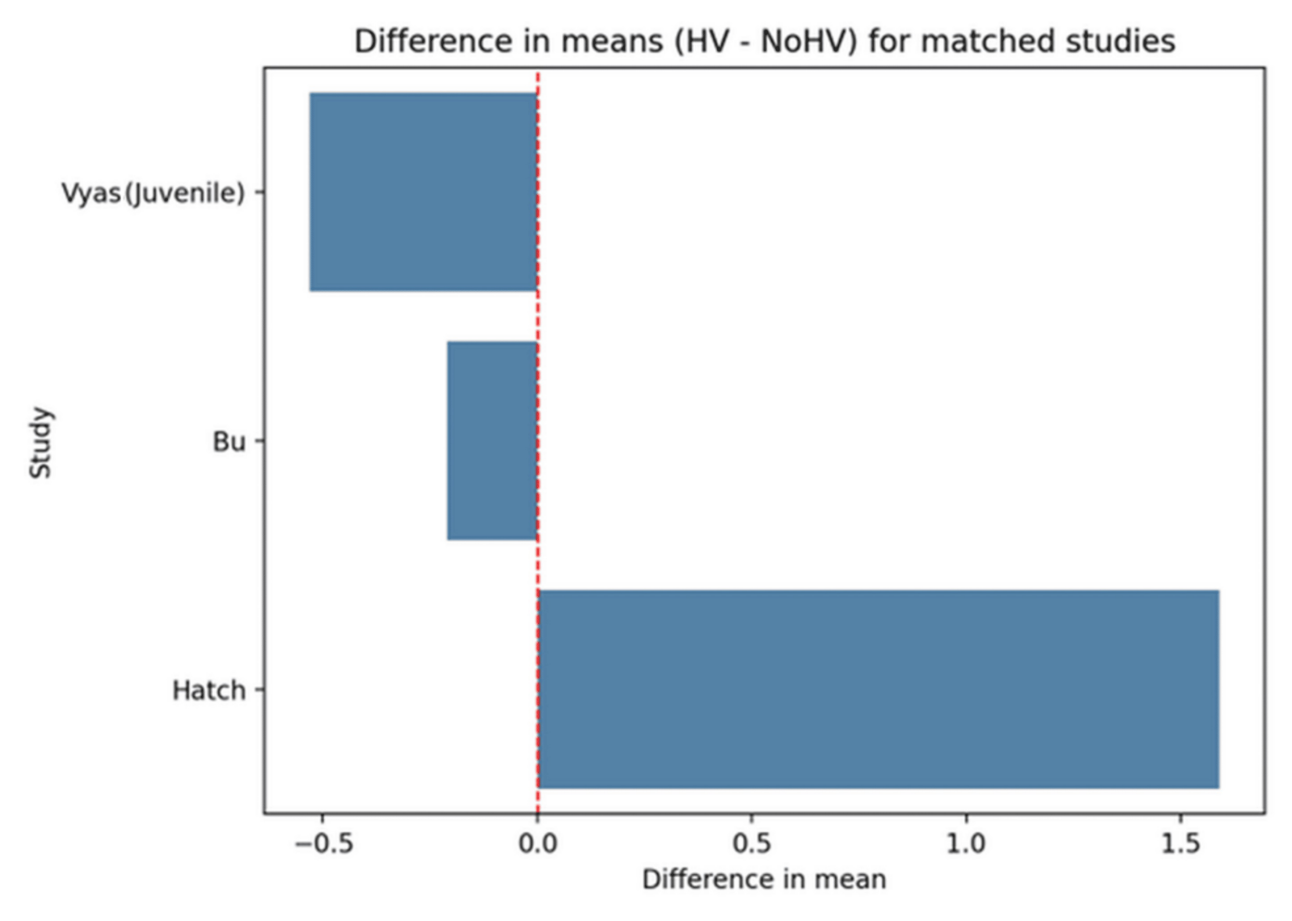
Mean difference in distal medial cuneiform angle (DMCA) between hallux valgus and control feet in matched studies

**Figure 3:**
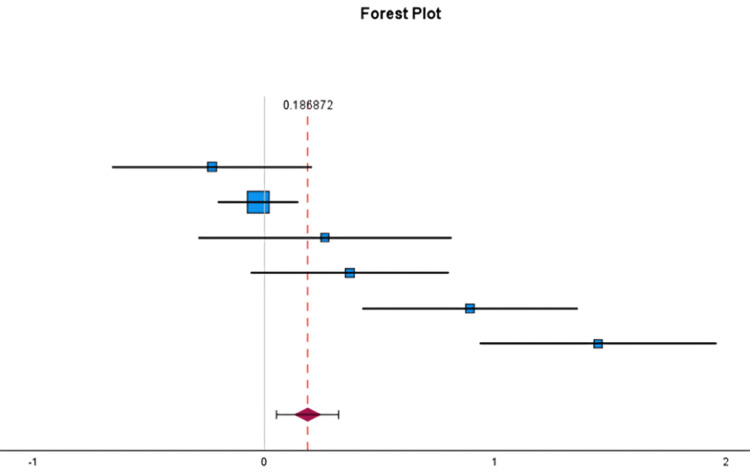
Forest plot of pooled Hedges’ g effect size for distal medial cuneiform angle (DMCA) comparing hallux valgus and control feet The blue square represents the effect size of each study. The red-dashed line indicates the overall effect size. The diamond shape represents the estimated overall effect size with its confidence interval.

The DMCA in our analysis, based upon four studies that had controls and met the inclusion criteria, demonstrated that the DMCA was higher in patients with HV (Figure 2). The overall Hedges' g effect size was only 0.19. However, this was statistically significant: p < 0.05 (0.05-0.32) (Figure 3).

Discussion

The term metatarsus primus varus, first described in 1924, refers to an oblique or varus alignment of the first metatarsal, historically considered a primary factor in the development of HV (HV). A varus first metatarsal alters motion at the first TMTJ, making it oblique rather than perpendicular to the long axis of the metatarsal shaft. If the medial cuneiform joint slope is a risk factor for HV, surgical correction, such as osteotomies of the medial cuneiform, could serve as an alternative to a Lapidus midfoot arthrodesis.

Lateral-based closing wedge osteotomies of the medial cuneiform, often combined with distal soft tissue realignment, have been used to correct HV [12]. Similarly, opening wedge osteotomies have been explored to modify the medial cuneiform slope [13]. Anatomical studies indicate that the first TMT joint is medially oblique in feet with HV [5].

An increased DMCA could theoretically predispose the first metatarsal to medial deviation into a varus position, thus contributing to the progression/development of HV.

**Figure 4:**
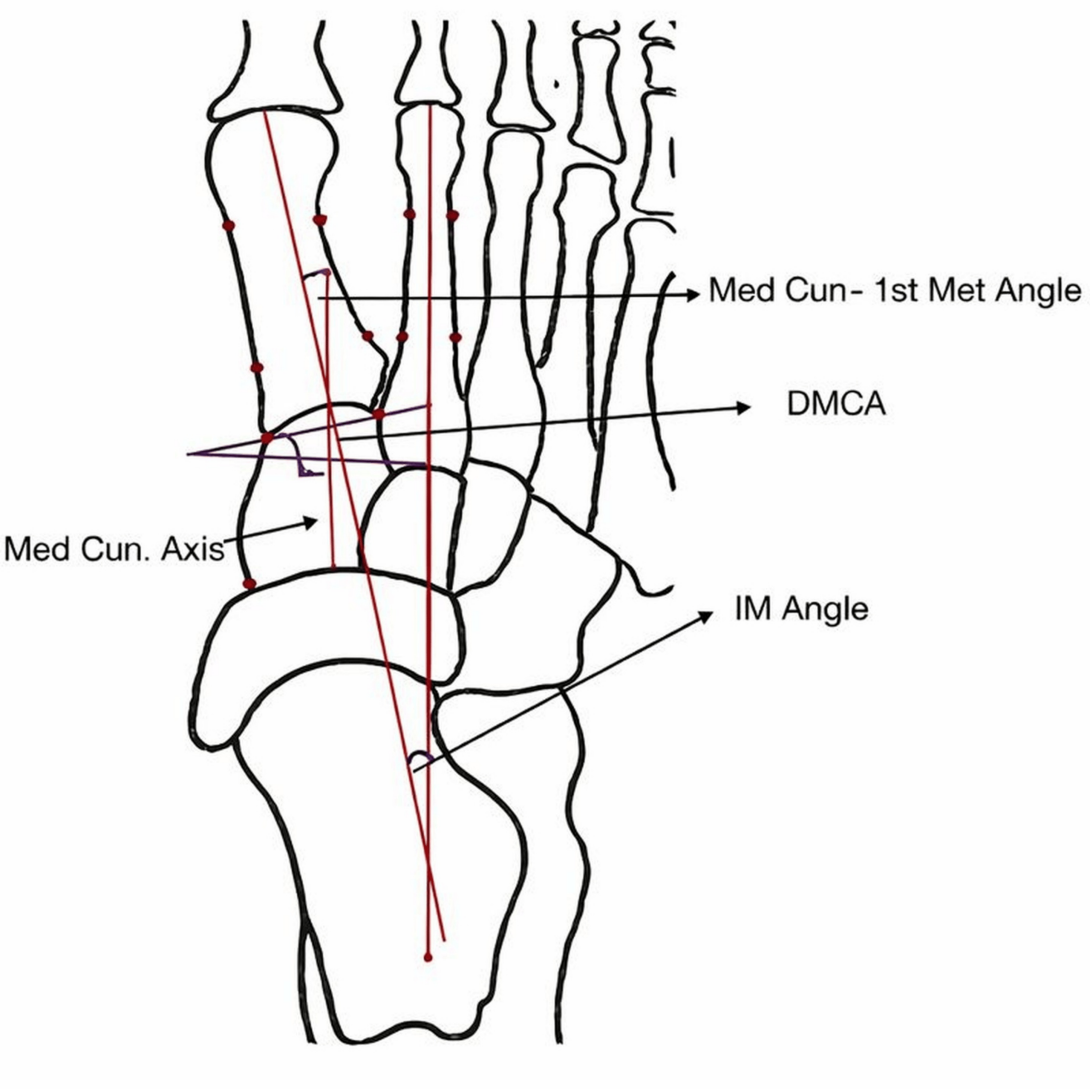
Radiographic measurement of the distal medial cuneiform angle (DMCA) using the medial cuneiform longitudinal axis, as described by Hatch et al. [10] The black irregular outline represents the medial cuneiform in a schematic view. The vertical black line denotes the mechanical axis (medial cuneiform axis). The lower blue horizontal line indicates the proximal joint orientation line (PJOL), and the upper oblique blue line represents the distal joint orientation line (DJOL). The red dots mark the reference points used to define the joint orientation lines. The distal medial cuneiform angle (DMCA) represents the deviation of the mechanical cuneiform axis (the angular relationship between the DJOL and the mechanical axis).

Hatch et al. [10] prospectively evaluated the DMCA in 25 adult patients, comparing those with an intermetatarsal angle (IMA) of less than 10° to patients with an IMA greater than 10°. Standardized weight-bearing dorsoplantar (DP) radiographs with a 15° dorsiflexion beam orientation were used. The study measured the DMCA and first intermetatarsal angle. The mean DMCA values were 20.69° in normal feet, 23.51° in moderate HV, and 20.41° in severe HV. Interrater reliability was high (over 0.72) for all measured angles. Their results suggested an inverse relationship between DMCA and HV severity, indicating that DMCA alone is not predictive of HV (Figure 4).

**Figure 5:**
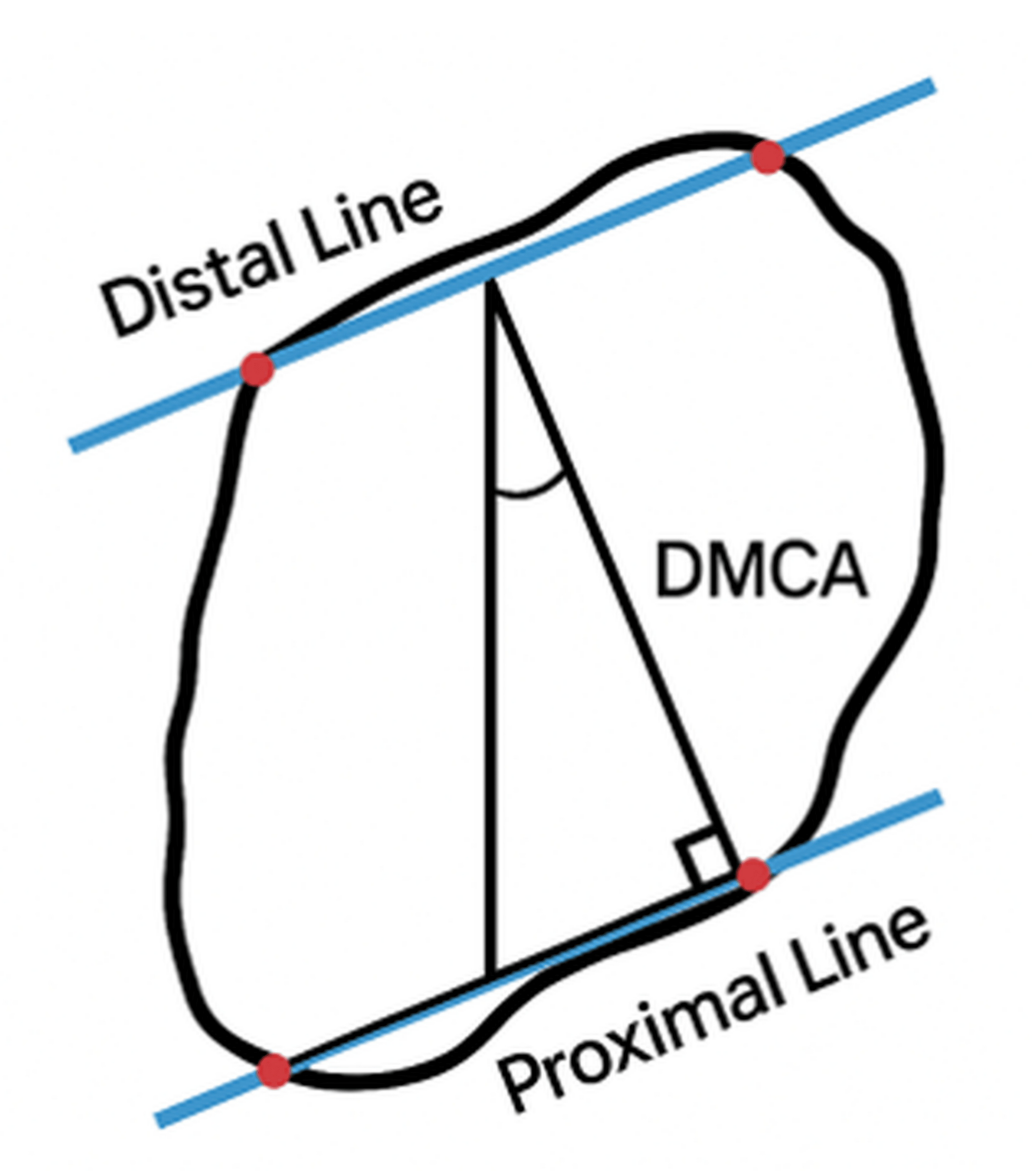
Radiographic measurement of the distal medial cuneiform angle (DMCA) using proximal and distal joint orientation lines, as described by Bu et al. [4] The distal line represents the distal joint orientation line, and the proximal line represents the proximal joint orientation line. The black vertical line denotes the mechanical axis. The distal medial cuneiform angle (DMCA) represents the deviation of the mechanical cuneiform axis. The red dots indicate the joint reference points.

Chen et al. [4] evaluated 679 feet in 538 patients (aged 18 and older) using 15° oblique dorsoplantar (DP) radiographs. They measured the HV angle (HVA), IMA, metatarsus adductus angle, first metatarsal-cuneiform angle, and DMCA. Additionally, the surface morphology of the first TMT joint (classified as flat or curved) was recorded (Figure 5).

In a study, 41% of participants were male, with ages ranging from 18 to 82 years (mean 36 years) [4]. The non-HV group comprised 155 patients (168 feet), of whom 100 (65%) were male, while the HV group included 383 patients (511 feet), with 131 males (33%). The distal cuneiform surface was four times more likely to be curved than flat, irrespective of HVA. The DMCA was significantly smaller in the non-HV group compared with the mild-to-moderate HV group (p < 0.05). However, only a weak negative correlation was observed between DMCA and both HVA and IMA, suggesting that DMCA is not a reliable predictor of HV severity.

**Figure 6:**
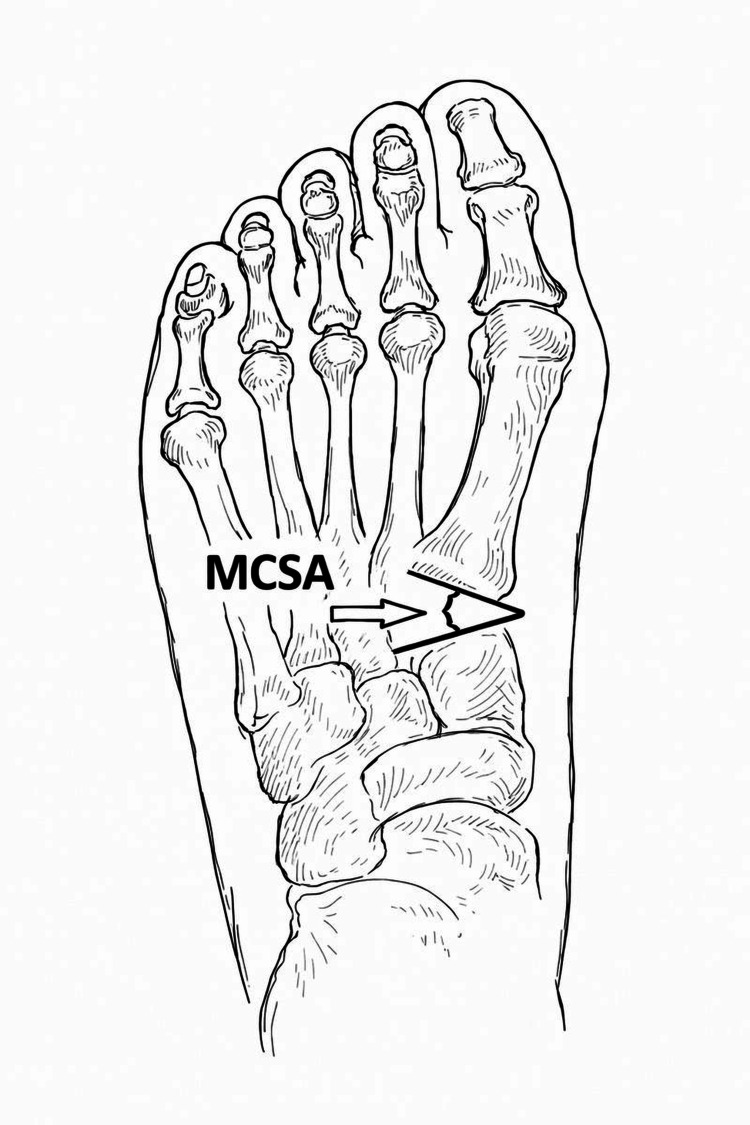
Radiographic measurement of the distal medial cuneiform angle (DMCA) using the medial cuneiform axis and metatarsal base reference line, as described by Erduran et al. [1] MCSA: Muscle cross-sectional area.

Erduran et al. [1] evaluated weight-bearing anteroposterior (AP) radiographs of 152 feet, categorizing them into four groups based on HV (HV) severity: no deformity, mild, moderate, and severe. The study assessed the first metatarsocuneiform angle (MCA), DMCA, and medial cuneiform lateral tilt angle (MCLTA) (Figure 6).

Statistically significant differences in the DMCA were observed between the no-deformity group (group 1) and both the mild (group 3, p = 0.034) and severe (group 4, p = 0.001) deformity groups. The mean DMCA also demonstrated significant differences across the groups:

Group 1/no deformity: 19.26° (SD 4.97)

Group 2/mild deformity: 22.54° (SD 5.62

Group 3/moderate deformity: 26.13° (SD 6.36)

Group 4/severe deformity: 32.17° (SD 5.85)

Significant differences were observed between group 1 and group 3 (p = 0.04) and between group 1 and group 4 (p = 0.023).

**Figure 7:**
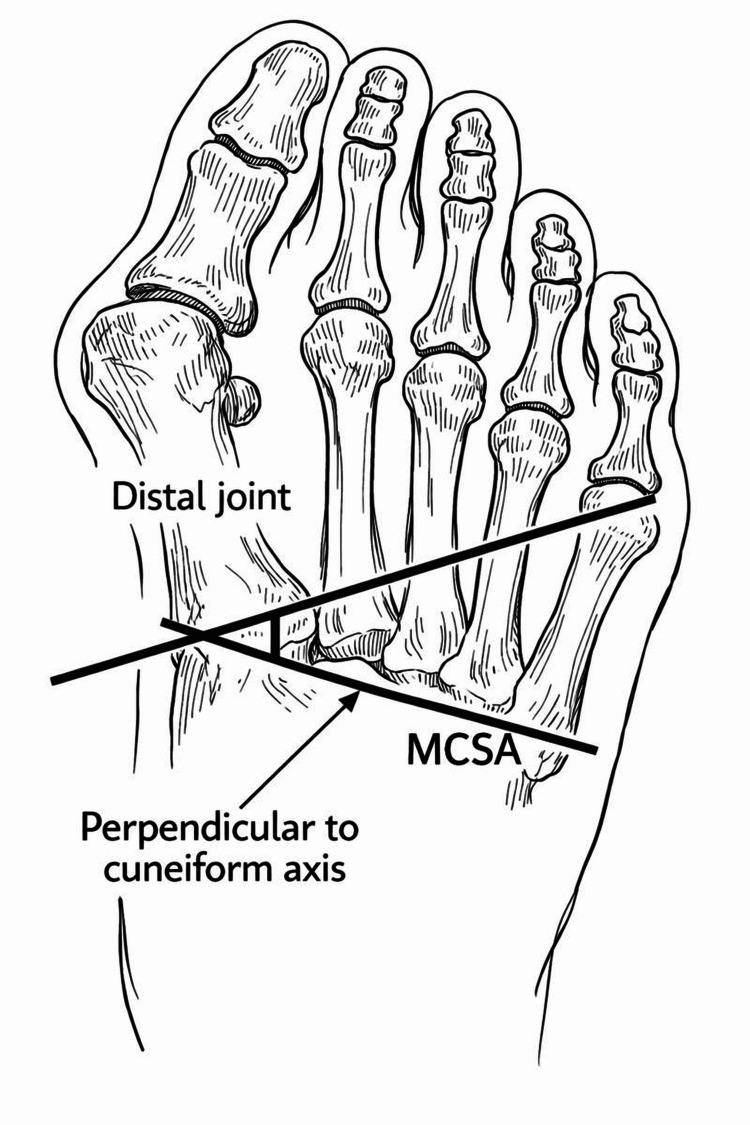
Radiographic measurement of the distal medial cuneiform angle (DMCA) using a perpendicular reference line to the distal joint surface, as described by Morio et al. [2] MCSA: Muscle cross-sectional area.

Vyas et al. [5] evaluated the role of medial cuneiform joint slope in JHV. The study included 46 feet from 29 patients with HV (mean age: 14.2 years; intermetatarsal angle > 10.8°) and 36 feet from 25 age-matched controls (mean age: 13.2 years). Measured parameters included the IMA, first metatarsal-medial cuneiform angle, first metatarsal-cuneiform angle, DMCA, and distal metatarsal articular angle. No statistically significant difference in DMCA was identified between the HV and control groups, suggesting that DMCA may not play a major role in the development of juvenile HV. Morio et al. [2] assessed 200 feet from 116 patients with HV (mean age: 68.9 years) using weight-bearing anteroposterior (AP) radiographs (Figure 7).

HV severity was classified as mild (HVA < 30°, IMA: 9°-13°), moderate (HVA: 30°-40°, IMA: 13°-20°), and severe (HVA > 40°, IMA > 20°); no control group without HV was included. The study evaluated the HVA, IMA, DMCA, and first-to-second cuneiform length. A total of 163 feet from 93 patients were analyzed, of whom 77 were female.

The mean DMCA was 23.9° (SD ± 6.0) in the mild group, 26.4° (SD ± 5.3) in the moderate group, and 30.2° (SD ± 4.3) in the severe group. The severe group demonstrated significantly higher DMCA values compared with both the mild and moderate groups (p < 0.001). The authors concluded that increasing DMCA may contribute to the development of more severe HV. However, the absence of a non-HV control group limits the interpretation of causality. The method used to define DMCA is measured as the angle between the distal joint orientation line of the medial cuneiform and a line perpendicular to a line connecting the midpoints of the distal and proximal joint surfaces.

**Figure 8:**
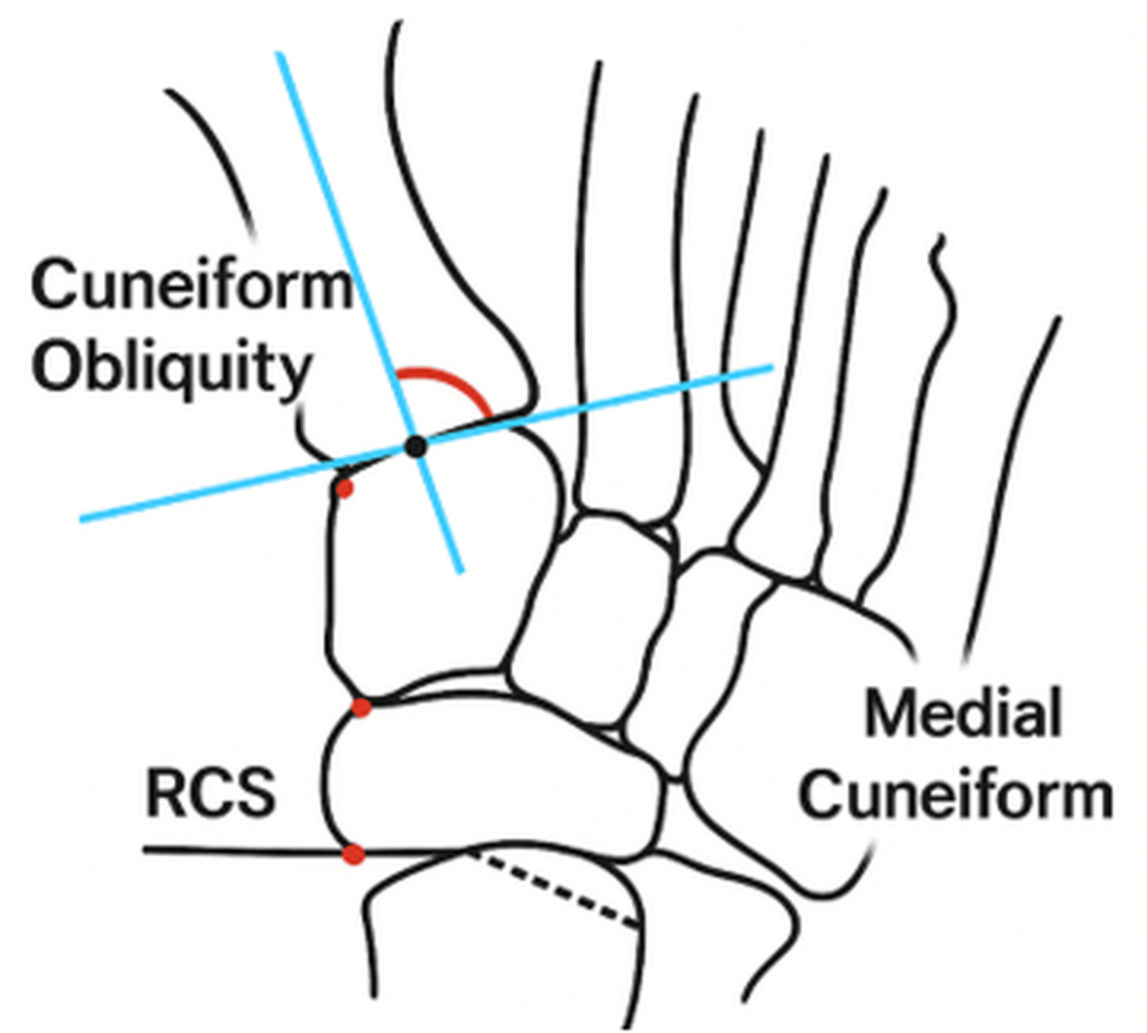
Radiographic assessment of medial cuneiform obliquity using the cuneiform transverse reference system, as described by Kaiser et al. [3]

Two studies assessed medial cuneiform obliquity using non-uniform angles: Kaiser et al. [3] evaluated weight-bearing radiographs of 93 feet from 57 JHV patients and 50 feet from 36 controls (Figure 8).

Measurements included the HVA, IMA, distal metatarsal articular angle (DMAA), Meary’s angle, and cuneiform obliquity (CO). As CO was referenced to the transverse axis of the cuneiform rather than its longitudinal axis, it was not included in the present analysis. A statistically significant increase in CO was observed between control subjects and those with mild-to-moderate JHV (8.7° vs. 12.6°, p < 0.001), with a moderate positive correlation between CO and IMA (r = 0.48).

**Figure 9:**
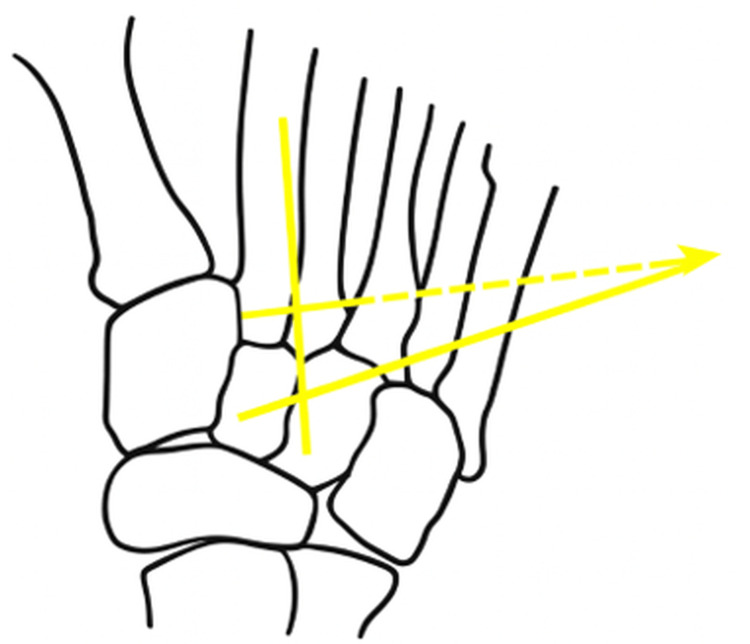
Radiographic measurement of the first metatarsocuneiform angle using metatarsal reference axes, as described by Patel et al. [1]

Patel et al. [11] assessed the first metatarsocuneiform angle (MCA) using nonstandard angles in 136 weight-bearing radiographs from patients with no history of foot trauma. In addition to MCA, the study measured IMA, HVA, tibial sesamoid position, Engel's angle, and two obliquity measures in the transverse and sagittal planes. No statistically significant difference in MCA was found between HV and non-HV groups (Figure 9).

**Figure 10:**
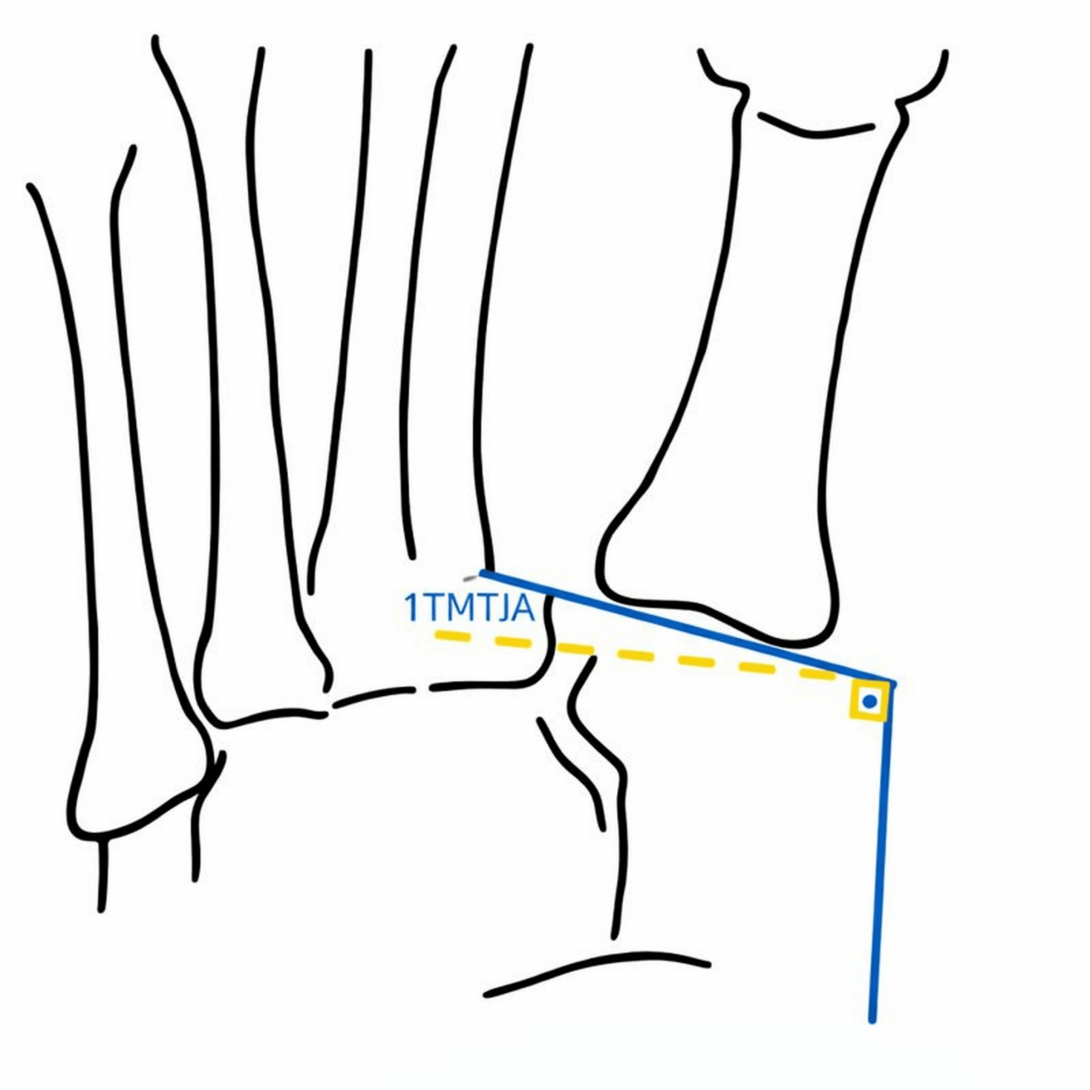
Radiographic measurement of the first tarsometatarsal joint angle (1TMTJA) under controlled foot and beam positioning, as described by Koury et al. [14]

Beam angulation may also contribute to variability in radiographic measurements. Koury et al. [14] evaluated 10 adult fresh-frozen cadaver specimens to assess the effect of foot positioning, specifically 0°, 5°, 10°, 15°, and 20° of dorsiflexion, plantarflexion, inversion, and eversion-on measurements of the first TMTJ angle and the morphology of the distal medial cuneiform articular surface (flat versus curved). Foot position was found to significantly influence the measured first TMTJ angle (p < 0.001), with a mean value of 22.9° (95% CI: 21.9-24.0). Increased dorsiflexion and inversion resulted in higher measured first TMTJ angles. Beam angulation was also shown to alter the apparent slope of the distal medial cuneiform. Given that HV deformity may destabilize the first ray and increase foot pronation, alterations in foot position and beam orientation during radiographic acquisition may further influence measured angular parameters, contributing to measurement variability and potential misinterpretation (Figure 10).

Beam angulation during radiographic acquisition can influence slope measurements. Erduran et al. measured the distal metatarsal slope rather than the base of the medial cuneiform; however, given that these structures are approximately parallel, these measurements were included in the final analysis. Hatch et al. and Bu et al. explicitly reported using 15° dorsoplantar weight-bearing (DP) views, whereas Kaiser et al. employed weight-bearing views without specifying beam angulation, which may have led to underestimation of slope measurements if standard anteroposterior views were used [3,4,10,15].

Although a statistically significant difference in DMCA was observed between the HV and control groups, this difference may lack clinical relevance given the small effect size. Our analysis demonstrated substantial heterogeneity (I² = 87.9%). This heterogeneity is likely attributable to differences in patient populations (juvenile versus adult), variation in HV severity (including mild, moderate, and severe DMCA subsets), differences in measurement techniques and radiographic beam angles, and variation in study design.

Clinical studies attempting to correct DMCA via opening wedge osteotomies have reported substantial recurrence rates [16-18], suggesting that additional factors, particularly soft tissue characteristics, may play a critical role. Importantly, variations in the DMCA are best interpreted in the context of first-ray stability rather than as an isolated morphological risk factor. DMCA alone cannot predict the presence of first-ray instability in HV. An increased DMCA may reduce intrinsic osseous stability at the first metatarsocuneiform joint, facilitating plantar-medial translation and rotational instability of the first ray under load. This may increase reliance on ligamentous and muscular restraints and contribute to progressive deformity in the presence of hindfoot pronation or soft tissue laxity.

Given the small effect size identified in this review, DMCA is unlikely to represent a primary causal factor in HV. Instead, it may act as an amplifying variable within a multifactorial biomechanical model. Surgically, an increased DMCA alone does not justify proximal or joint-sacrificing procedures; however, in patients with demonstrable first-ray instability or recurrent deformity, DMCA morphology may help explain persistent instability and support the use of stabilizing medial column procedures rather than distal correction alone.

Limitations

Adult and juvenile populations were not directly compared, and studies did not report interobserver or intraobserver reliability for measurements of the medial cuneiform longitudinal axis or consistently specify the use of dorsoplantar radiographic views. Beam direction is known to influence angular measurements, and all studies were retrospective in design. Two studies with notable findings were discussed but excluded from the analysis, and CT-based or finite element studies were not considered. This review does not establish causality, and variation in DMCA may represent adaptive changes secondary to HV rather than a primary causal factor, although weak negative correlations in some studies suggest this is unlikely to be a major effect. Pronation and first-ray instability were not assessed. Interpretation is further limited by the constraints of two-dimensional radiography, variability in measurement definitions, and the absence of randomized or longitudinal data.

## Conclusions

Based on three included studies, patients with HV demonstrated a statistically significant increase in the DMAA compared with controls (26.4° vs 22.5°). However, the small effect size (0.19) limits its clinical relevance. In addition, no studies have examined associations with non-radiographic factors, such as first-ray instability, which are important for treatment decisions. Stronger relationships have been reported for other radiographic parameters, and the usefulness of the DMCA is further constrained by radiographic projection variability and the three-dimensional nature of the TMTJ. Overall, although DMCA shows a minor statistical association with HV, it is unlikely to serve as a reliable independent predictor of deformity severity or progression.

## Figures and Tables

**Figure 11:**
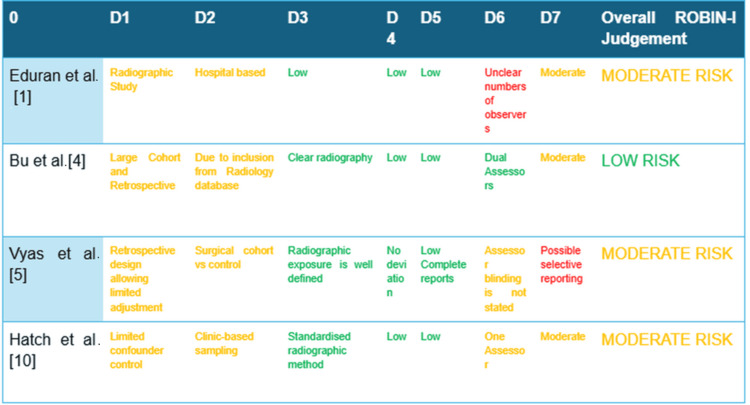
Table representing risk of bias Key: D1 - Bias due to confounding. D2 - Bias in selection of participants. D3 - Bias in classification of exposure. D4 - Bias due to deviation from intended exposure. D5 - Bias due to missing data. D6 - Bias in measurements of outcomes. D7 - Bias in selection of reported results. Risk of bias judgment: Low risk - Green. Some concerns/Moderate - Yellow. High risk - Red.

## Appendices

**Table 5:** Erduran et al., 2016 [1] Inclusion criteria: Normal feet and mild, moderate, and severe hallux valgus deformities. Exclusion criteria: Congenital foot anomalies, previous foot surgery, previous tarsal or metatarsal fractures, and diabetic foot disease.

Group	n	Men (n)	Women (n)	Age range (years)	Mean age ± SD (years)
Normal	40	8	32	17–63	45 ± 15.86
Mild hallux valgus	43	6	37	16–72	51 ± 17.62
Moderate hallux valgus	36	3	33	24–66	49 ± 14.31
Severe hallux valgus	33	5	28	31–78	52 ± 17.44

**Table 6:** Morio et al., 2024 [2]

Variable	Total	Mild	Moderate	Severe
Patients (n)	93	–	–	–
Feet (n)	163	63	36	64
Sex (M/F)	16/77	Sep-54	May-31	Aug-56
Age (years)	68.9 ± 10.2	67.5 ± 12.4	71.1 ± 9.8	71.7 ± 9.8
Hallux valgus angle (°)	≥15	15–30	30–40	≥40
Height (m)	1.54 ± 0.62	1.54 ± 0.69	1.53 ± 0.52	1.54 ± 0.64
Weight (kg)	51.2 ± 8.7	50.4 ± 8.6	52.1 ± 7.2	51.4 ± 9.9
BMI (kg/m²)	21.7 ± 3.3	21.3 ± 3.2	22.3 ± 3.2	21.7 ± 3.4

**Table 7:** Kaiser et al., 2018 [3] JHV: Juvenile hallux valgus.

Characteristic	Normal	JHV
Patients, n	36	57
Feet, n	50	93
Age, years	13.2 ± 2.5	14.1 ± 2.1
Sex: male, n (%)	15 (30%)	23 (25%)
Sex: female, n (%)	35 (70%)	70 (75%)

**Table 8:** Bu et al., 2023 [4]

Category	Description	
Study period	January 2018–February 2021	
Age range, years	18–82	
Inclusion criteria	Diagnosis of hallux valgus; weight-bearing anteroposterior foot radiographs	
Exclusion criteria	Congenital foot deformity; history of foot or ankle surgery; severe arthritis with unidentifiable joint space	
Total participants	538 patients (679 feet)	
Sex: male, n (%)	222 (41%)	
Sex: female, n (%)	316 (59%)	
Characteristics	Normal Group	Hallux Valgus Group
Patients, n	155	383
Feet, n	168	511
Sex: male, n (%)	100 (65%)	131 (33%)
Sex: female, n (%)	55 (35%)	252 (67%)

**Table 9:** Vyas et al., 2010 [5]

Category	Control (Normal) Group	Hallux Valgus Group
Subjects, n	25 patients	29 patients
Feet, n	36	46
Mean age, years	13.2	14.2
Eligibility criteria	Exclusion of gross foot deformities; no known diagnoses affecting midfoot or forefoot structure or function; age <12 years or >16 years	Diagnosis of idiopathic juvenile hallux valgus (JHV); open physes of the feet; no gross unrelated foot deformities or underlying diagnoses; patients who underwent surgical correction of symptomatic bunion deformity

**Table 10:** Hatch et al., 2016 [10]

Category	Description/Value
Total participants	50
Normal feet (Group A)	25
Hallux valgus feet (Group B)	25
Moderate hallux valgus	15 (60%)
Severe hallux valgus	10 (40%)
Age criterion	≥18 years
Study period	September 2014–January 2015
Sex	Not specified
Group	Definition
Normal feet	No clinical evidence of hallux valgus and intermetatarsal angle (IMA) < 10°
Moderate hallux valgus	IMA 10°–15°
Severe hallux valgus	IMA > 15°
General selection criteria	Presence of measurable IMA and no history of previous first-ray surgery

**Table 11:** Patel et al., 2019 [11]

Characteristics	Value
Subjects, n	99
Sex: male/female, n	28/71
Feet, total n	136
Left-sided feet, n	66
Right-sided feet, n	70
Mean age, years	47.43 ± 14.98
Age range, years	18–78

**Table 12:** Koury et al., 2019 [14]

Characteristics	Value	
Specimens	10 above-knee thawed fresh-frozen cadavers	
Sex: male/female, n	05-May	
Age range at death, years	54–88	
Mean age at death, years	79.9	
Characteristics	Control (Normal Feet)	Hallux Valgus Feet
Feet, n	23	23
Patients, n	17	13
Male, n	2	2
Female, n	15	11
Mean age, years	52.26 ± 3.9	55.3 ± 14.4
Hallux valgus angle (HVA) criteria	<15°	<40°

**Table 13:** Doty et al., 2014 [15]

Characteristics	Value		
Specimens	39 cadaveric feet		
Sex, n	Male: 12; Female: 22; Unknown: 5		
Age range, years	45–95		
Mean age, years	79		
Age unknown, n	6		
Exclusion criteria	Previous foot surgery; significant pathologic abnormalities of the foot other than hallux valgus		
Hallux Valgus Severity	Feet, n (%)	Mean HVA (°)	Mean 1–2 IMA (°)
Normal	5 (13%)	11.6	7.6
Mild	12 (31%)	15.8	9.6
Moderate	18 (46%)	26.5	12
Severe	4 (10%)	45.8	17

**Table 14:** Li et al., 2024 [16]

Characteristics	Control (Normal Feet)	Hallux Valgus Feet
Feet, n	23	23
Patients, n	17	13
Male, n	2	2
Female, n	15	11
Mean age, years	52.26 ± 3.9	55.3 ± 14.4
Hallux valgus angle (HVA) criteria	<15°	>40°
